# Prescribed medicines among pregnant women attending health facilities in Eritrea: patterns, safety, and determinants

**DOI:** 10.3389/fphar.2026.1806253

**Published:** 2026-07-21

**Authors:** Nuru Abdu, Saleh Idrisnur, Alemseghed Goitom Tesfagaber, Dawit G. Weldemariam, Tomas Tewelde, Eyasu H. Tesfamariam, Mulugeta Russom

**Affiliations:** 1 Medicine Information Services Unit, Pharmacy Services Division, Department of Medical Services, Ministry of Health, Asmara, Eritrea; 2 Product Evaluation and Registration Unit, National Medicines and Food Administration, Ministry of Health, Asmara, Eritrea; 3 Debub Zonal Pharmaceutical Services, Ministry of Health, Mendefera, Eritrea; 4 Department of Pharmacy, Hazhaz Zonal Referral Hospital, Ministry of Health, Asmara, Eritrea; 5 Gash-Barka Zonal Pharmaceutical Services, Ministry of Health, Barentu, Eritrea; 6 Department of Statistics, Biostatistics and Epidemiology Unit, College of Sciences, Mai-Nefhi, Eritrea; 7 Eritrean Pharmacovigilance Center, National Medicine and Food Administration, Ministry of Health, Asmara, Eritrea

**Keywords:** determinants, Eritrea, Pregnancy and Lactation Labeling Rule (PLLR), pregnant women, prescription pattern, safety

## Abstract

**Background:**

Prescribing unsafe medications during pregnancy poses significant risks for adverse maternal and fetal outcomes. This nationwide study assessed medication utilization patterns, safety risk classifications, and associated determinants among pregnant women in Eritrea.

**Methods:**

A cross-sectional study was conducted in 32 health facilities across Eritrea from October to December 2024. Data were collected through patient interviews, prescription reviews, and medical record abstractions. Medication safety was evaluated by translating Pregnancy and Lactation Labeling Rule (PLLR) narrative summaries into three clinical tiers (safe, cautious, and contraindicated) and comparing them with the legacy United States Food and Drug Administration (US-FDA) pregnancy risk categories. Descriptive statistics and logistic regression analyses were performed using IBM SPSS version 26.0 to identify factors associated with exposure to potentially unsafe medications.

**Results:**

A total of 740 pregnant women were included, with a mean (SD) age of 26.95 (5.75) years. A total of 1,031 medicines were prescribed, with a mean of 1.39 (SD = 0.65) medications per prescription. According to the legacy US-FDA system, the prevalence of prescriptions containing potentially risky medicines (Categories C and D) was 4.2% (95% CI: 3.0, 5.9). Under the PLLR framework, 96.7% of the medications were classified as “safe,” 2.4% as “cautious,” and 0.9% as “contraindicated.” The most common “Cautious” medications were metronidazole (n = 10) and nitrofurantoin (n = 3), while “Contraindicated” agents primarily included diclofenac (n = 5) and ciprofloxacin (n = 2). While the agreement between the two systems was high (96.7%), the Cohen’s kappa (kappa = 0.486) indicated moderate concordance. Type of healthcare facility (AOR = 2.38; 95% CI: 1.11, 5.12), presence of chronic illness (AOR = 12.56; 95% CI: 3.52, 44.88), and first-trimester gestational age (AOR = 3.39; 95% CI: 1.28, 8.99) were significantly associated with exposure to potentially unsafe medicines under the legacy system, while urban residence (AOR = 2.82; 95% CI: 1.23, 6.49) was a significant determinant under the PLLR framework.

**Conclusion:**

Prescribing practices during pregnancy were largely characterized by low polypharmacy and a predominant reliance on medications classified as lower-risk under both frameworks. However, the transition to the PLLR framework provides a more precise risk assessment, addressing the ambiguity of legacy categories. Targeted interventions are needed for high-risk groups, particularly women with chronic illnesses, those in early pregnancy, and urban residents.

## Introduction

Pregnancy is a time of significant physiological change, creating complex challenges for clinicians who must manage pre-existing conditions and new illnesses while selecting safe medications (Pinheiro and Stika, 2020; Eke et al., 2023). Despite the medical recommendation to avoid drugs during gestation due to the potential for adverse fetal and maternal effects, this is often impractical (Ross et al., 2015; Wesley et al., 2021). Many women require essential drug treatment for chronic disorders, such as hypertension, epilepsy, and diabetes mellitus, or for acute pregnancy-related symptoms, making prescribing an inherent benefit-risk evaluation (Famiglietti et al., 2025; Geresu et al., 2020). Therefore, drugs are vital for maintaining the health of both the mother and the fetus (Sewell et al., 2022; Edyedu et al., 2025).

A substantial lack of reliable safety data hinders the decision-making process. Ethical and safety concerns routinely exclude pregnant women from pre-marketing clinical trials (Sewell et al., 2022; van der Graaf et al., 2018). Consequently, most medications are marketed without a definitive safety profile established for human pregnancy (Wood et al., 2019). The historical tragedy of thalidomide in the 1960s serves as a constant reminder of the teratogenic potential of prescription drugs (Russell et al., 1962). While some major teratogens are known, the risk of subtle or minor fetal developmental effects remains largely undetermined for the majority of drugs (Parisi et al., 2011).

To assist healthcare providers in minimizing risks, various systems have been developed to categorize medication safety during pregnancy, including the Swedish, ADEC, and the United States Food and Drug Administration (US-FDA) pregnancy risk category (Addis et al., 2000; Law et al., 2010; Sannerstedt et al., 1996). The US-FDA system (Categories A, B, C, D, and X) classifies drugs based on their risk profile, with Categories D and X indicating clear fetal risk (Law et al., 2010; Department of Health and Human Services FDA, 2008). However, this framework frequently led clinicians to misinterpret the recommendations as a grading system of risk rather than a categorization of available data (Byrne et al., 2020). The FDA revised its labeling system in 2015 with the more narrative Pregnancy and Lactation Labeling Rule (PLLR) (Department of Health and Human Services FDA, 2008). The PLLR is preferred over the legacy system because it replaces oversimplified letter categories, often misinterpreted as a safety scale, with detailed narrative summaries of available human and animal data (Danesh and Murase, 2015).

Despite the limited safety information, medication use in pregnancy is highly prevalent globally. High rates of drug utilization, ranging from 27% to 93%, have been recorded in developed countries (Lupattelli et al., 2014). Similarly, studies in developing nations like Ethiopia have reported considerable use of prescribed medicines, alongside instances of unsafe prescribing practices (Alema et al., 2020; Daw et al., 2011). Irrational drug use in these settings is compounded by low maternal literacy, insufficient current information among prescribers, a lack of standard guidelines, and poor antenatal care access (Eser and Çelik, 2022).

Given the potential for teratogenicity and other serious consequences, assessing the extent and safety profiles of prescribed drugs is a crucial public health measure to promote rational use of medicine in this population. However, there is a critical lack of local data on drug utilization patterns and their safety in pregnant women in Eritrea. Thus, this study aimed to assess the pattern of drug utilization and the safety risk classification of prescribed medicines and its associated risk factors among pregnant women in selected health facilities in Eritrea.

## Methods

### Study design and setting

A facility-based cross-sectional study using a quantitative approach was conducted among pregnant women attending antenatal care (ANC) clinics in Eritrea. Eritrea operates a three-tier healthcare system: primary (health stations, health centers, and community hospitals), secondary (second-contact hospitals and zonal referral hospitals), and tertiary (national referral hospitals). Maternal health services, including prenatal and childbirth care, are provided at all public health facilities, with institutional delivery strongly encouraged through maternity waiting homes and 24-h emergency obstetric care services.

Antenatal care in the country follows a standardized national protocol aligned with World Health Organization (WHO) guidelines, focusing on the early detection of complications, tetanus toxoid immunization, nutritional supplementation (iron and folic acid), and screening for infections such as HIV, syphilis, and hepatitis. While routine prenatal care is largely managed by midwives and associate nurses at lower-tier healthcare facilities, high-risk pregnancies are referred to regional and national referral hospitals where specialized obstetric care is available.

The study was carried out across 32 health facilities, consisting of health centers and community hospitals. These facilities are distributed across all six administrative regions of Eritrea: Maekel (six facilities), Anseba (five), Gash-Barka (ten), Semenawi Keih Bahri (four), Debubawi Keih Bahri (one), and Debub (six). Data collection was performed over a three-month period from October to December 2024.

### Target population

The source population comprised all pregnant women who visited the selected health facilities in Eritrea for antenatal care or other services during the study period. The target population included pregnant women of any gestational age who were prescribed at least one medication at the facility and who consented to participate. Women who were not prescribed any medications were excluded.

### Sample size determination

Sample size was computed by considering the finite population correction factor: n = Z^2^pq/d^2^. The total sample size (n) was calculated using the following assumptions: expected proportion of pregnant women who are given potential risk medicine (p) and those who are not given a potential risk medicine (q) were taken as 0.5, Z statistic for 95% level of confidence (Z = 1.96) and margin of error (d) of 0.05. Considering the above assumptions, the initial sample size was 385.

$$n_{2}=n\;\left(385\right)\;x\text{ design effect }\left(1.75\right)=674$$

After consideration of 10% non-response rate, the final sample was 749.

### Sampling technique

Two-stage stratified cluster sampling was used to select the pregnant women from the first antenatal care visitors of the health facilities that render relevant services in each region. Each region was considered a stratum. At first stage, the health facilities were selected using probability proportionate to size (PPS). At the second stage, the pregnant women were selected from the health facilities using systematic random sampling. The sampling interval (K) for each facility was determined by the ratio of the average monthly patient flow, as recorded in the national Health Management Information System (HMIS), to the allocated sample size. A random starting point was then utilized to initiate the selection process.

### Sample allocation

The overall sample size was proportionally allocated to each region. The distribution of pregnant women by region is shown in Supplementary Material 1.

### Data collection tool and approach

A structured questionnaire and a standardized data abstraction format, developed based on a review of similar published studies (Alema et al., 2020; Leke et al., 2018; Devkota et al., 2016), were used to collect data. The data collection instrument consisted of four sections. Section A includes socio-demographic and background characteristics of the patients, such as age, residence, occupation, educational level, marital status, ethnicity, religion, chronic illness, and pregnancy stage. Section B intends to record information of the prescribed medicine(s) from the patient’s prescriptions for medicine utilization pattern analysis. Section C intends to record information from patients’ clinical cards, such as current diagnosis and history of chronic illness. Section D was aimed at analyzing the safety of the prescribed medicines.

The data collectors explained purposes of the study to the selected participants, and those who gave consent were enrolled. To trace the socio-demographic and background characteristics, an interview was conducted for each respondent. Then, information contained in their prescriptions were recorded, and their clinical cards were assessed to document their medical history including co-morbid conditions, diagnosis of the last visit, and drug history. All the prescribed medicines were assigned a US-FDA pregnancy risk category (A, B, C, D and X) by the research team.

Besides, the conversion of descriptive narratives from the PLLR framework into three analytical tiers, safe, cautious, and contraindicated, was performed by two researchers who evaluated the narrative evidence (human versus animal data) and trimester-specific warnings to ensure that the categorization reflected both current regulatory standards and clinical best practices. All the obtained data were documented, and no follow-ups were made.

### Variables

Pregnancy potential risk medicine was considered as a dependent variable, whereas age, residence, type of healthcare facility, presence of chronic illness, and pregnancy status were considered as independent variables.

### Outcome measures

The safety of prescribed medications was evaluated using both the legacy US-FDA pregnancy categories (A, B, C, D, and X) and the PLLR risk summaries. Drugs categorized as C, D, or X were considered to have pregnancy potential risk for use. The pattern of medication use was assessed by identifying the most frequently prescribed drugs and therapeutic classes according to the Anatomical Therapeutic Chemical (ATC) classification system.

To enable a structured comparison, narrative data were extracted from the Drugs@FDA database (U.S. Food and Drug Administration, 2024) and verified via the Drugs.com Professional Database (Drugs.com, 2024), which provides synchronized access to the most current FDA-approved labeling. The PLLR risk summaries were classified into three clinical tiers:

- **Safe:** Medications where the PLLR narrative, supported by human or animal data, indicates no evidence of fetal risk, or the summary explicitly identifies the agent as a ‘preferred’ treatment option.
- **Cautious:** Medications where the narrative highlights specific precautions, such as trimester-dependent risks (e.g., avoidance near term or in the first trimester), but allows for use if the clinical benefit to the mother outweighs the potential fetal risk.
- **Contraindicated:** Medications where the FDA-approved labeling explicitly states the drug should be avoided or is contraindicated due to established evidence of human fetal harm or significant risk of developmental toxicity.

### Data quality assurance

Face and content validity of the data collection instrument was confirmed through expert review by a panel of pharmacists and an epidemiologist. To maintain data quality, orientation was provided to the data collectors on the study protocol, ethical considerations, and data collection tools. A pre-test was conducted in selected health facilities that are not part of the study. It was aimed at checking the data collection tool’s appropriateness, completeness, flow of questions, skip patterns, as well as familiarization of data collectors, and approximation of the time for completion of the data collection tool. As per the experiences from the pre-test, the data collection tool was revised and used for the actual data collection.

Moreover, to minimize potential discrepancies in the PLLR-derived risk classifications, two researchers independently categorized each identified medication. Discrepancies particularly those involving drugs with complex or trimester-dependent narratives, were resolved through a formal consensus-building session. Final classifications were recorded only upon unanimous agreement following a thorough re-review of the primary FDA-approved professional labeling. This verification process ensured that the findings were based on validated regulatory summaries rather than subjective interpretation.

The study was reported in line with the Strengthening the Reporting of Observational Studies in Epidemiology (STROBE) statement (Von et al., 2014).

### Ethical approval

Ethical approval was obtained from the Ministry of Health (MOH) Research Ethics and Protocol Review Committee (reference number: 17/09/2023). Besides, permission was obtained beforehand from the Ministry of health regional medical offices. Study participants were informed about the study’s purpose, and written informed consent was obtained from each respondent. All the data obtained was kept confidential, and only anonymized data was used solely for the study’s purposes. This study conforms to the principles outlined in the Declaration of Helsinki (World Medical Association, 2013).

### Statistical analysis

The collected data were double-entered on CSPro (version 7.3) (U.S. Census Bureau and ICF International, 2020) to minimize the keying errors. The entered data were exported to IBM SPSS (version 26.0) (IBM Corp, 2019) for statistical analysis.

To account for the complex survey design, statistical analysis was performed using the SPSS Complex Samples module. A sampling plan was defined with regions as strata and healthcare facilities as primary sampling units (PSUs). Sampling weights were calculated as the inverse of the selection probability at both stages and used to produce population-representative estimates. Standard errors and 95% confidence intervals were adjusted for the design effect using Taylor series linearization.

Descriptive summaries of quantitative socio-demographic variables were presented as mean (Standard Deviation (SD)) for normally distributed data. Primarily, factors associated with the prescribing of potentially unsafe medications (Categories C, D, or X) were assessed using bivariate logistic regression (CSLOGISTIC) derived from the US-FDA pregnancy risk categories. Furthermore, factors associated with the clinical risk of prescribed medications derived from the PLLR approach, classified into three ordered categories (safe, cautious, and contraindicated), were analyzed using Ordinal Logistic Regression (PLUM procedure).

Variables demonstrating significance at the bivariate level (p ≤ 0.15) were retained for multivariable analysis. Crude Odds Ratios (COR) and Adjusted Odds Ratios (AOR) with 95% Confidence Intervals (CI) were computed to assess the magnitude and direction of the associations. Statistical significance in the final multivariable models was defined as p ≤ 0.05. For the ordinal models, the proportional odds assumption was verified using the test of parallel lines, and model fit was evaluated using Nagelkerke R-square.

To mitigate sparse-event bias and ensure sufficient statistical power for multivariable logistic regression, medications under legacy US-FDA Categories C and D were pooled into a single composite binary dependent variable defined as ‘pregnancy potential risk’. This consolidation is justified both statistically, to stabilize maximum likelihood estimates given low absolute cell frequencies, and clinically, as both categories represent therapeutic choices where potential risks necessitate an explicit, deliberate benefit-risk justification prior to prescribing.

To evaluate the level of agreement and ensure consistency between the historical US-FDA pregnancy risk categories and the newly derived PLLR-based risk summaries, a concordance analysis was performed using Cohen’s Kappa statistic for categorical variables.

## Results

### Socio-demographic and background characteristics of the study population

During the study period, data collectors approached 749 pregnant women. Nine women were excluded either because they were not prescribed any medications or because they were unwilling to participate, resulting in a final sample of 740 pregnant women included in the study.

A total of 740 pregnant women were included in the analysis. The mean age of participants was 26.95 years (SD = 5.75), with the largest proportion falling within the 20–34 years (80.0%, n = 592) age group. A majority of women resided in rural areas (59.2%, n = 437). Most of the pregnant women attended health centers (58.9%, n = 436). Nearly all women (97.8%, n = 724) reported no chronic illness. Among the small proportion with chronic conditions, asthma was the most frequently reported (25.0%, n = 4), followed by hypertension and diabetes (each 12.5%, n = 2). In terms of gestational age, most of the women (44.1%, n = 326) were in the third trimester. A detailed socio-demographic and background characteristics are depicted in Table 1.

**TABLE 1:** Socio-demographic and background characteristics of the participants (n = 740).

Variable	Category	Frequency	Percentage
Age (M = 26.95, SD = 5.75)	Less than 20	52	7.0
	20 to 34	592	80.0
	35 or above	96	13.0
Zoba	Maekel	117	15.8
	Anseba	107	14.5
	Debub	149	20.1
	Semenawi Keih Bahri	93	12.6
	Debubawi Keih Bahri	19	2.6
	Gash-Barka	255	34.5
Residence	Urban	301	40.8
	Rural	437	59.2
Facility level	Health center	436	58.9
	Community hospital	304	41.1
Occupation	Employed	72	9.8
	Unemployed	666	90.2
Chronic illness^1^	Hypertension	2	12.5
	Diabetes	2	12.5
	Asthma	4	25.0
	Renal Failure	1	6.3
	Others*	7	43.8
	No chronic illness	724	97.8
Religion	Christian	349	47.2
	Muslim	391	52.8
Gestational age	First trimester	117	15.8
	Second trimester	296	40.1
	Third trimester	326	44.1

M: Mean, SD: Standard Deviation, Others* include cardiac problems, epilepsy, human immune-deficiency virus, psychosis, and thyroid *disorder,* ^1^Specific chronic illnesses (e.g., asthma, hypertension) are calculated using the sub-population of women with a chronic illness (n=16) as the denominator

### Medicine utilization pattern

A total of 1,031 medicines were prescribed with a mean of 1.39 (SD = 0.65) medicines per prescription. Moreover, the minimum and maximum number of medicines per prescription were one and 4, respectively (Table 2). The majority of the prescriptions contained one medicine (68.4%, n = 506). The most commonly prescribed medicines were ferrous sulfate and folic acid (53.5%, n = 552), amoxicillin (22.2%, n = 229), paracetamol (9.7%, n = 100), and aluminum hydroxide/magnesium hydroxide (antacid) (3.9%, n = 40).

**TABLE 2:** Medicine utilization pattern.

Number of drugs per prescription (M = 1.39, SD = 0.65)	Frequency	Percentage
1	506	68.38
2	184	24.86
3	42	5.68
4	8	1.08
Total	740	100

M, Mean; SD, Standard Deviation.

Out of 921 indications, antenatal care (n = 513), urinary tract infection (n = 205), anemia (n = 44), acute pain (n = 31), and gastritis (n = 26) were the most common reasons for prescription (Table 3).

**TABLE 3:** Common indications for the prescribed medicine (n = 921).

Indication	Frequency	Percentage	ICD-10	Description
Preventive supplementation for antenatal care	513	55.7	Z36.9	Antenatal screening, unspecified
UTI	205	22.3	N39.0	Urinary tract infection, site not specified
Anemia	44	4.8	D64.9	Anemia, unspecified
Acute pain	31	3.4	G89.1	Acute pain, not elsewhere classified
Gastritis	26	2.8	K29.70	Gastritis, unspecified, without bleeding

UTI, Urinary Tract Infection.

### Safety of prescribed medicines using the US-FDA pregnancy risk category

The prevalence of pregnancy potential risk was 4.2% (95% CI: 3.0, 5.9). Based on the US-FDA pregnancy risk classification, the majority of prescribed medicines were Category A (56.6%) and Category B (40.1%). A small proportion of medicines were categorized as Category C (2.9%) and Category D (0.4%). No Category X medications were prescribed to any participant in this cohort.

Omeprazole was the most frequently prescribed Category C medicine (n = 8) (Table 4). Category D exposures involved diclofenac use during the third trimester (n = 2), enalapril (n = 1), and phenobarbitone (n = 1).

**TABLE 4:** Medicines involved in pregnancy potential risk according to the US-FDA.

Medicine	ATC code	Frequency	Risk category
Omeprazole	A02BC01	8	C
Ciprofloxacin	J01MA02	2	
Diclofenac	M02AA15	2	
Salbutamol	R03CC02	2	C
Diclofenac (in third trimester)	M02AA15	2	D
Enalapril	C09AA02	1	D
Phenobarbitone	N03AA02	1	D

### Determinants of pregnancy-related medicine risk

At bivariate analysis, type of healthcare facility (*p* = 0.036), the presence of chronic illness (*p* < 0.001), and first-trimester gestational age (*p* = 0.041) were significantly associated with exposure to pregnancy-risk medicines (Table 5).

**TABLE 5:** Determinants of maternal medication risk categories under the US- FDA pregnancy risk category.

Variable	Category	Bivariate analysis		Multivariable analysis	
		COR (95% CI)	*p-*value	AOR (95% CI)	*p-*value
Age	-	0.99 (0.94, 1.06)	0.986	-	-
Residence	Rural	0.59 (0.28, 1.22)	0.158	-	-
	Urban	*Ref*	​	​	​
Facility level	Health center	*Ref.*	-	*Ref.*	-
	Community hospital	2.22 (1.06, 4.69)	0.036	2.38 (1.11, 5.12)	0.026
Chronic illness	Yes	8.95 (2.70, 29.63)	<0.001	12.56 (3.52, 44.88)	<0.001
	No	*Ref*	-	*Ref*	-
Gestational age	First trimester	2.63 (1.04, 6.65)	0.041	3.39 (1.28, 8.99)	0.014
	Second trimester	1.22 (0.51, 2.91)	0.655	1.43 (0.57, 3.56)	0.444
	Third trimester	*Ref*	*-*	*-*	*-*

AOR, Adjusted Odds Ratio; CI, Confidence Interval; COR, Crude Odds Ratio.

Exploratory multivariable logistic regression indicated that pregnant women who attended community hospitals were two times more likely to be exposed to potentially risky medicines than those who attended health centers (AOR = 2.38; 95% CI: 1.11, 5.12; *p* = 0.026). Furthermore, pregnant women with chronic illness were twelve times more likely to be exposed to potentially risky medicines than those without chronic illness (AOR = 12.56; 95% CI: 3.52, 44.88; *p* < 0.001) (Table 5). Pregnant women in the first-trimester gestational age were more likely to be exposed to potentially risky medicines than those in the third trimester gestational age (AOR = 3.39; 95% CI: 1.28, 8.99; *p* = 0.016).

### Safety of prescribed medicines using the PLLR framework

Of the 1,031 medicines evaluated, 96.7% were classified as safe and categorized as preferred for clinical use (Table 6). In contrast, medicines identified as contraindicated or requiring avoidance represented only 0.9% of the total. The evidence base was primarily derived from human studies (74.4%) or a combination of human and animal data (25.3%), while trimester-specific risks were identified in a minority of cases (3.4%).

**TABLE 6:** Descriptive analysis of PLLR risk and clinical recommendations (n = 1,031).

Variable	Category	Frequency	Percentage
PLLR risk summary	Safe	997	96.7
	Cautious	25	2.4
	Contraindicated	9	0.9
Evidence type	Human	767	74.4
	Animal	3	0.3
	Both	261	25.3
Trimester specific risk	Yes	35	3.4
	No	996	96.6
Clinical recommendation	Preferred	997	96.7
	Avoid	9	0.9
	Benefit outweighs risk	25	2.4

Within the “Cautious” category, Metronidazole (n = 10) and Nitrofurantoin (n = 3) were the most frequently observed medications. Similarly, among those listed as “Contraindicated,” Diclofenac (n = 5) and Ciprofloxacin (n = 2) appeared most often (Table 7).

**TABLE 7:** Clinical risk summary of common cautions and contraindicated medicines under the PLLR framework.

Medicine name	PLLR risk summary	Frequency	Evidence type	Trimester-specific risk	Clinical description
Metronidazole	Cautious	10	Human/Animal	Yes	Avoid high-dose regimens in 1st trimester
Nitrofurantoin		3	Human	Yes	Avoid at term (38–42 weeks) due to haemolytic risk
Artesunate		2	Animal	Yes	Limited data for the 1st trimester use
Cotrimoxazole		2	Human/Animal	Yes	Folate antagonist; avoid in 1st trimester and at term
Diclofenac	Contraindicated	5	Human	Yes	Contraindicated after 30 weeks (ductus arteriosus)
Ciprofloxacin		2	Animal	No	Potential for arthropathy; alternatives preferred
Enalapril		1	Human	Yes	Contraindicated in the 2nd and 3rd trimesters
Ibuprofen		1	Human	Yes	Avoid in late pregnancy (renal/cardiac risks)

### Determinants of maternal medication risk categories under the PLLR framework

In the bivariate analysis, residence (*p* = 0.002) and healthcare facility level (*p* = 0.037) were significantly associated with medication risk categories (Table 8).

**TABLE 8:** Determinants of maternal medication risk categories under the PLLR Framework.

Variable	Category	COR (95% CI)	*p*-value	AOR (95% CI)	*p*-value
Age	-	0.99 (0.93, 1.05)	0.770	-	-
Residence	Urban	3.34 (1.56, 7.16)	0.002	2.82 (1.23, 6.49)	0.014
	Rural	*Ref.*	-	*Ref.*	-
Facility level	Health center	0.46 (0.23, 0.96)	0.037	0.70 (0.32, 1.55)	0.386
	Community hospital	*Ref.*	-	*Ref.*	-
Chronic illness	Yes	3.40 (0.75, 15.38)	0.111	2.91 (0.61, 13.80)	0.179
	No	*Ref.*	-	*Ref.*	-
Gestational age	First trimester	1.92 (0.76, 4.82)	0.166	-	-
	Second trimester	1.11 (0.49, 2.51)	0.803	-	-
	Third trimester	*Ref.*	-	-	-

AOR, Adjusted Odds Ratio; CI, Confidence Interval; COR, Crude Odds Ratio.

After adjusting for potential confounders, exploratory multivariable analysis indicated that pregnant women residing in urban areas had nearly a three-fold increase in the odds of being prescribed medications in higher risk tiers compared to those in rural areas (AOR = 2.82, 95% CI: 1.23, 6.49, *p* = 0.014) (Table 8).

### Concordance between FDA pregnancy categories and PLLR risk summaries

A total of 1,031 medications were analyzed to evaluate the degree of agreement between the historical FDA letter-based categories (A, B, C, D, and X) and the PLLR risk summaries. The overall observed agreement between the two systems was 96.7%. However, when adjusting for chance agreement, the Cohen’s kappa coefficient was kappa = 0.486 (*p* < 0.001), representing moderate concordance.

Analysis of the agreement across specific risk classifications revealed high stability for low-risk medications but lower consistency for intermediate and high-risk drugs. For medications previously classified as Category A or B, 98.7% (n = 984/997) remained within the “Safe” PLLR summary. In contrast, only 34.5% (n = 10/29) of drugs formerly designated as Category C were classified as “Cautious” under the PLLR framework. For the high-risk group (Categories D and X), 60.0% (n = 3/5) maintained a “Contraindicated” status under the new system.

Reclassification occurred in both upward and downward directions across the dataset. Upward risk revision (escalation) was noted in 1.3% (n = 13/997) of drugs previously labeled “Safe” (A/B), which moved to the “Cautious” category. Notably, 20.7% (n = 6/29) of drugs formerly labeled as Category C were escalated to “Contraindicated.”

Conversely, downward risk revision (de-escalation) was observed most frequently in Category C, where 44.8% (n = 13/29) of medications were reclassified as “Safe” under PLLR narratives. Besides, 40.0% (n = 2/5) of medications previously categorized as D or X were reclassified into the ‘Cautious’ category.

## Discussion

This nationwide cross-sectional study assessed prescribing patterns, safety, and determinants among pregnant women in Eritrea. Using both legacy US-FDA pregnancy risk categories and the PLLR, findings indicate a cautious prescribing environment with low polypharmacy and predominant reliance on lower-risk medications. Most prescriptions adhered to safe practices; however, critical vulnerabilities were identified, particularly the use of contraindicated agents among high-risk groups such as women with chronic illnesses, those in the first trimester, and urban residents. These findings underscore the need for targeted interventions to optimize maternal and fetal outcomes.

Data indicated a relatively low prevalence of chronic illnesses (2.2%), which should be interpreted within the context of the current healthcare landscape. Similar findings have been reported in other low-resource settings, where low prevalence may reflect early-stage screening, underdiagnosis of asymptomatic conditions, and limited access to specialized longitudinal care (Firoz et al., 2022). In contrast, studies from higher-income settings show that chronic conditions such as hypertension and diabetes are frequently managed throughout pregnancy (Jiang et al., 2022).

The observed mean of 1.39 medicines per prescription reflects restrained prescribing consistent with reports from other low-resource settings. National antenatal guidelines emphasize daily iron and folic acid supplementation, which concentrates prescribing on essential supplements and acute pregnancy indications such as urinary tract infections. Facility-based prescription data may not capture over-the-counter medications, traditional herbal remedies, or privately obtained supplements, which are commonly reported in similar cultural contexts and can lead to underestimation of total medication exposure (Anand et al., 2023). Regional differences in healthcare access and medication availability likely contribute to variation in the number of agents prescribed across various settings (Anand et al., 2023; World Health Organization, 2020).

Under the updated PLLR narrative framework, the vast majority of medications identified in this study were classified as lower-risk, which broadly aligns with the legacy US-FDA Categories A and B. Only a small proportion of prescriptions fell into the “Cautious” or “Contraindicated” classifications. This suggests a generally careful clinical approach to prescribing that prioritizes both maternal and fetal safety.

Among the agents requiring cautious prescribing under the PLLR framework, metronidazole and nitrofurantoin were the most frequently observed. Metronidazole is widely utilized to treat anaerobic bacterial and protozoal infections; while current human data do not indicate a robust association with an increased teratogenic risk, clinical guidelines recommend avoiding high-dose regimens during the first trimester to mitigate potential theoretical risks (Burtin et al., 1995). Similarly, nitrofurantoin is generally considered safe for treating urinary tract infections in early pregnancy; however, its use is typically cautioned or avoided near term due to the potential risk of inducing hemolytic anemia in the newborn (Bookstaver et al., 2015; American College of Obstetricians and Gynecologists’ Committee on Obstetric Practice, 2017).

The prescription of non-steroidal anti-inflammatory drugs (NSAIDs), specifically diclofenac and ibuprofen, was also identified and classified as contraindicated during late pregnancy. Exposure to NSAIDs in the third trimester raises significant clinical concerns due to their well-documented potential to disrupt prostaglandin synthesis, which can cause premature closure of the fetal ductus arteriosus and impair fetal renal function, ultimately leading to oligohydramnios (Bloor and Paech, 2013; Østensen, 1998; Antonucci et al., 2012). For the management of pain during pregnancy, safer alternatives such as paracetamol at the lowest effective dose for the shortest duration are generally preferred, given their established safety profiles compared with NSAIDs, which must be strictly avoided in late gestation (Nunge et al., 2025; Shah et al., 2015; Black et al., 2019).

Similarly, the use of ciprofloxacin and enalapril necessitates strict clinical scrutiny. Ciprofloxacin, classified under restricted use, is generally avoided during pregnancy because of concerns regarding potential articular cartilage toxicity and arthropathy observed in animal studies; consequently, safer alternatives like beta-lactam antibiotics are prioritized unless bacterial resistance or severe intolerance necessitates its use (Bookstaver et al., 2015; Acar et al., 2019; Loebstein et al., 1998). Enalapril, an angiotensin-converting enzyme inhibitor, is strictly contraindicated in the second and third trimesters due to its well-established association with profound fetopathy, including impaired fetal renal development and subsequent severe complications (Cooper et al., 2006; Bullo et al., 2012; Buawangpong et al., 2020). When antihypertensive management is required, switching to safer alternatives such as labetalol or methyldopa is strongly recommended.

Under the legacy US-FDA pregnancy risk category framework, maternal chronic illness, clinical setting, and gestational age were significantly associated with the likelihood of exposure to high-risk medications. Specifically, pregnant women with chronic illnesses are significantly more likely to receive potentially hazardous medications out of therapeutic necessity, as managing complex maternal conditions often outweighs theoretical fetal risks (Brown et al., 2026; Dunlop et al., 2008). These findings should be interpreted with caution due to the limited absolute number of high-risk exposures. Furthermore, exposure to high-risk agents is notably elevated during the first trimester, the critical period of fetal organogenesis, a vulnerability frequently compounded by delayed pregnancy confirmation and late initiation of antenatal care (Winterstein et al., 2024; Dibaba et al., 2024). The care setting also plays a major role; women attending community hospitals experience higher exposure than those at health centers, reflecting greater case complexity and specialized care needs.

However, under the updated PLLR framework, residence emerged as an additional significant determinant, with pregnant women residing in urban settings demonstrating higher odds of being prescribed medications in higher risk tiers compared to those in rural areas. This urban-rural disparity may reflect broader differences in diagnostic capacity, specialized disease burden, or the availability of advanced pharmacological treatments at urban community hospitals. Collectively, these findings underscore the urgent need to enhance pre-conception counseling to optimize drug regimens, facilitate early access to antenatal care, and strengthen routine pharmacovigilance to minimize avoidable fetal risks.

In this study, the moderate concordance observed between historical US-FDA categories and the contemporary PLLR risk summaries indicates that while both frameworks align for well-established, low-risk therapies, the transition provides a distinct clinical assessment for medications with more complex safety profiles (Pernia and DeMaagd, 2016). While drugs formerly designated as Category A or B demonstrate high stability and remain reliable indicators of safety, the substantial volatility identified within Category C highlights its long-standing critique as an ambiguous “catch-all” classification (Ramoz and Patel‐Shori, 2014). By reclassifying a significant proportion of these medications into more definitive “Safe” or “Contraindicated” summaries, the PLLR framework addresses critical data gaps and the inherent clinician confusion in the previous system, which often lacked the granularity required for informed risk-benefit counseling (Blattner et al., 2016). These shifts illustrate that the current narrative-based approach offers a more precise reflection of emerging human evidence and clinical context, fostering individualized decision-making that the simplistic letter-based system could not effectively support (Pernia and DeMaagd, 2016; Blattner et al., 2016).

Monitoring medication use during pregnancy is crucial for safeguarding maternal and fetal health. An integrated framework, guided by evidence-based protocols and ongoing provider education, supports informed and safer prescribing within antenatal care. While prescribing practices in this study predominantly involved medicines classified as lower-risk, the occasional use of contraindicated agents highlights the need for continued prescriber training on trimester-specific risks. These findings emphasize strengthening pregnancy pharmacovigilance and updating guidelines, to improve understanding of medication safety.

This study has limitations that should be considered when interpreting the findings. First, the cross-sectional design precludes assessment of specific maternal or neonatal outcomes following medication exposure, limiting the ability to draw definitive conclusions about the clinical impact of observed prescribing patterns. Moreover, the analysis relied on prescription records, which may not fully reflect actual patient adherence or the use of over-the-counter medicines or herbal remedies, both of which are common during pregnancy. Because the study only included women who received at least one prescription, these findings may not be generalizable to the broader population of pregnant women in Eritrea who do not seek institutional care. Besides, while the legacy FDA categories were used for historical comparison, their limitations in clinical nuance are acknowledged.

## Conclusion and recommendation

This study indicates that medication utilization during pregnancy was characterized by limited polypharmacy and a predominance of agents generally classified as lower-risk for obstetric use. However, the shift from legacy US-FDA letter categories to the modernized PLLR narrative framework reveals critical nuances in risk assessment. While the overall exposure to high-risk medications is low, the identification of contraindicated agents, particularly among women with chronic conditions, those in their first trimester, and residents of urban areas, highlights persistent vulnerabilities within antenatal prescribing practices. The moderate concordance between the two systems underscores the value of the PLLR in resolving the clinical ambiguity historically associated with ‘Category C’ drugs, many of which were de-escalated to ‘Safe’ upon detailed narrative review.

To mitigate avoidable risks, it is essential to strengthen early antenatal care, integrate routine medication reviews, and provide targeted professional development for healthcare providers on trimester-specific pharmacotherapy. Ultimately, developing context-specific treatment guidelines and establishing pregnancy drug-exposure registries will be vital to fostering evidence-based decision-making and optimizing maternal and fetal health outcomes in Eritrea.

## Data Availability

The original contributions presented in the study are included in the article/Supplementary Material, further inquiries can be directed to the corresponding author.
