# Supplementary material for: Prescribed medicines among pregnant women attending health facilities in Eritrea: patterns, safety, and determinants
Source: Front Pharmacol. 2026 Jul 21;17:1806253. doi: 10.3389/fphar.2026.1806253 (PMC13433617; doi:10.3389/fphar.2026.1806253)
Supplement: Supplementary file 1 [file Table1.docx]

**Supplementary material 1.** Distribution of pregnant women visitors across the zoba

| **Zoba** | **Estimated number of pregnant women visitors in two months** | **Samples taken** |
| --- | --- | --- |
| Maekel | 3542 | 118 |
| Anseba | 3290 | 109 |
| Gash Barka | 7736 | 257 |
| Semenawi Keih Bahri | 2734 | 91 |
| Debubawi Keih Bahri | 534 | 18 |
| Debub | 4735 | 157 |
| **Total** | **22,571** | **749** |
